# The use of Multi-Sensory Environments with autistic children: Exploring the effect of having control of sensory changes

**DOI:** 10.1177/13623613211050176

**Published:** 2021-10-24

**Authors:** Katy L Unwin, Georgina Powell, Catherine RG Jones

**Affiliations:** 1 Cardiff University, UK; 2 La Trobe University, Australia

**Keywords:** autism spectrum disorders, education services, interventions – psychosocial/behavioural, sensory impairments

## Abstract

Multi-Sensory Environments (also called sensory or Snoezelen^®^ rooms) are specialised spaces that contain equipment to modify the environment. They are commonly used in special-needs schools with autistic children, but empirical investigation into how to best use Multi-Sensory Environments with autistic children has been limited. Based on converging evidence that autistic children may benefit from having control, we tested 41 autistic children (8 female) aged 4–12 years (mean = 8 years, standard deviation = 2.05 years) who used the Multi-Sensory Environment both with and without control over the sensory changes. Behavioural coding of video data showed that having control was associated with increased attention and reduced repetitive motor behaviours, sensory behaviours, activity levels, stereotyped speech and vocalisations. Social behaviour, anxiety, positive affect and arousal were not significantly affected by condition. Our findings demonstrate that how a Multi-Sensory Environment is used can affect autistic children’s behaviours. They also suggest that providing control of sensory changes to autistic children may help create better conditions for learning.

**Lay abstract:**

Multi-Sensory Environments (also called sensory or Snoezelen^®^ rooms) are rooms that contain equipment which can create light, sound and touch experiences. Multi-Sensory Environments are often used with autistic children, particularly in schools, but there is no evidence for how best to use them. We investigated whether having control over the sensory equipment in the Multi-Sensory Environment affected how a group of 41 (8 female) autistic children aged 4–12 years behaved. We found that when autistic children could control the sensory equipment, they paid more attention and performed fewer repetitive and sensory behaviours. They also used less stereotyped speech, produced fewer vocalisations and showed lower levels of activity. Other behaviours were not affected. Our findings demonstrate that how a Multi-Sensory Environment is used can impact behaviour and that providing control of sensory changes to autistic children may help create better conditions for learning.

## Introduction

Multi-Sensory Environments (MSEs; also known as a sensory or Snoezelen^®^ rooms) are specialised spaces that contain equipment to modify the sensory environment across the modalities. They are used internationally (e.g. Bozic, 1997; J. Chan & Chien, 2017; Cuvo et al., 2001) and are common in special-needs schools. Their widespread use with autistic children is likely to relate to the differences in sensory processing experienced by autistic people, which comprise part of the core autism symptomatology (American Psychiatric Association, APA, 2013). Although MSEs are widely used with autistic pupils, there is a clear need for further evidence-based research to support their use (Botts et al., 2008; Cameron et al., 2019; Lotan & Gold, 2009). Previous work has demonstrated that the way MSEs are used can affect outcomes (Fava & Strauss, 2010; Glenn et al., 1996; Hill et al., 2012). For example, Fava and Strauss (2010) reported a reduction in stereotyped behaviours in their autistic and intellectual disabled (ID) adult participants when the MSE session was unstructured compared to when it was structured. Furthermore, Hill et al. (2012) reported greater engagement in the MSE when their ID participants were provided with high levels of caregiver attention compared to low levels. There is also evidence from a small-scale, descriptive study (*n* = 6) reporting that autistic children may show improvement in aspects of sensory functioning after using an MSE (Mey et al., 2015). However, no study to date has specifically investigated how to use the MSE to facilitate benefits for autistic children.

Educational practitioners have reported that being in an MSE can facilitate behavioural change for autistic children and this behaviour change can lead to improved opportunities for learning (Unwin et al., 2021). Particularly, practitioners believed that the child being able to have control of the sensory environment was a mechanism through which behaviour change occurred. Preference for environmental predictability by autistic people is reported in both qualitative (Ashburner et al., 2013; Robertson & Simmons, 2015) and questionnaire (Fujino et al., 2019) studies. Differences in the ability to predict environmental stimuli are also central to many recent frameworks put forward to understand autistic characteristics, such as Bayes and predictive coding (e.g. Palmer et al., 2017; Pellicano & Burr, 2012; Powell et al., 2016; Van De Cruys et al., 2014). Broadly, these theories suggest that autistic individuals may be relatively more driven by bottom-up sensory information than top-down predictions based on prior experience. This means that the world is more surprising and less predictable, which could lead to increased cognitive load and feelings of ‘sensory overload’. Restrictive repetitive behaviours (RRBs), a core characteristic of autism, are potentially a way of increasing the predictability of sensory stimulation. Furthermore, cognitive accounts emphasise a greater intolerance of uncertainty in autistic people (Boulter et al., 2014), which is heightened by sensory sensitivities (Boulter et al., 2014; Wigham et al., 2015). Increasing control over the sensory environment within the MSE could therefore relieve both the perceptual discomfort and psychological distress from uncertainty, thereby supporting behaviour change and creating better conditions for learning or receiving therapeutic intervention.

Interventions to control sensory stimulation in everyday environments have shown benefits for autistic children and adults, including the use of headphones (e.g. Ikuta et al., 2016; Pfeiffer et al., 2019; Rowe et al., 2011) and modification in lighting (e.g. Kinnealey et al., 2012). Across the studies, these adaptations led to a range of improvements in attention (Kinnealey et al., 2012; Pfeiffer et al., 2019; Rowe et al., 2011), meeting individualised goals (Ikuta et al., 2016), mood and classroom performance (Kinnealey et al., 2012), and reductions in anxiety and challenging behaviours (Pfeiffer et al., 2019). Kinnealey et al. (2012) also report some improvement in social interaction, although only after several weeks of intervention. These findings are consistent with teacher reports that increasing autistic children’s control over the classroom environment helps them control unwanted sensory experiences, and that it is the uncontrollable and unpredictable nature of sensory stimuli that causes the most difficulty for their learning (Jones et al., 2020).

Control of the MSE also affords the child an opportunity to better satisfy their sensory needs (e.g. Baillon et al., 2002). The sensory profiles and needs of autistic individuals are marked by heterogeneity (e.g. Leekam, Nieto, et al., 2007) and can affect anxiety (Boulter et al., 2014), RRBs (Boyd et al., 2010) and social behaviour (Hilton et al., 2007). Therefore, in having control of the MSE the autistic child could benefit from a tailored and more pleasant sensory environment, which in turn may have a positive effect on their ability to cope with uncertainty (Wigham et al., 2015).

In summary, MSEs are widely used with autistic children in the absence of empirical data that could guide their use. However, reports from practitioners (Unwin et al., 2021) suggest that optimal use of the MSE may come from providing autistic children with control over sensory changes. In the first study of its kind, we examined the effect of having control of the MSE on the behaviours, cognition and arousal levels of 41 autistic children aged 4–12 years. In the Active-Change condition, participants had control over the MSE equipment, and in the Passive-Change condition, the MSE equipment changed automatically without their input. We used observational coding methods to record behaviours previously identified by practitioners as outcomes of MSE use for autistic children (Unwin et al., 2021). These included social communication (including, speech, rapport, social behaviours, gestures and mimicry), repetitive motor behaviours (RMBs), sensory behaviours, anxiety, positive affect and attention. In addition, we measured activity level and heart rate variability (HRV), which provided an objective measure of arousal. It was hypothesised that having control of the MSE equipment would have a positive effect on behaviour and arousal compared to not having control.

## Method

### Participants

The final sample included 41 autistic children (8 female) aged 4–12 years (mean (M) = 8 years, standard deviation (SD) = 2.05 years). Originally, 44 took part but three were fully excluded from all analyses due to data loss from technical reasons (i.e. missing observational video for one condition) or not being able to access the study because of not coping with the 3-h testing period. Recruitment was through the Wales Autism Research Centre’s Facebook page and recruitment register. For inclusion, the child had to be aged 4–11 years (one participant turned 12 before testing), with a clinical diagnosis of autism, no diagnosis of another developmental disorder, and no significant hearing, visual or mobility differences. Finally, they needed to be able to work one-to-one with a previously unknown adult in the MSE.

Autism diagnosis was confirmed through laboratory administered Autism Diagnostic Observation Schedule (ADOS-2, Lord et al., 2012) assessments, with 40 participants having scores commensurate with their diagnosis (M = 7.8, SD = 1.45). The ADOS-2 video was missing for one participant, meaning scoring could not be completed. The child with the missing ADOS video scored 24 on the parent-completed Social Communication Questionnaire (SCQ; Berument et al., 1999), which is above the autism cut-off score (of >15; Rutter et al., 2003). Therefore, they were retained in the sample. The mean score on the SCQ for all 41 children was M = 24.8 (SD = 6.48).

The Wechsler Abbreviated Scale of Intelligence (WASI-II; Wechsler, 2011) was used to measure cognitive ability in verbally fluent participants aged 6 and over (*n* = 19). Those over 6 years old but not verbal (*n* = 8) received the two non-verbal intelligence quotient (NVIQ) sub-scales of the WASI-II. The Wechsler Preschool and Primary Scale of Intelligence (WPPSI-IV; Wechsler, 2012) was administered to those under the age of 6 (*n* = 8). Where participant fatigue or difficulty engaging prevented administration on the day of testing, a follow-up session was arranged at the participant’s school (*n* = 11). However, six participants were not able to access cognitive ability assessments on either occasion, resulting in cognitive ability data for 35 participants (WASI-II both full-scale intelligence quotient (FSIQ) and NVIQ = 27; WPPSI-IV = 8, Table 1).

**Table 1.** Cognitive ability data (*n* = 35) from the Wechsler Abbreviated Scale of Intelligence and Wechsler Preschool and Primary Scale of Intelligence assessments.

	*n*	Range	M	SD
Verbal intelligence quotient (VIQ)	19	59–126	94.32	19.74
Non-verbal intelligence quotient (NVIQ)	35	46–142	90.43	24.23
Full-scale intelligence quotient (FSIQ)	19	69–128	96.95	15.75

SD: standard deviation.

All parents provided written informed consent. Ethnic and socioeconomic data were not recorded for these participants. The experimental manipulation (i.e. provision of control) and the outcomes measured were developed following research interviews and surveys with practitioners working with autistic children in MSEs (Unwin et al., 2021). However, the autistic community was not directly consulted in the development of the study. Ethical approval for this study was granted by the School of Psychology Ethics Committee, Cardiff University.

### Materials and procedure

**Figure 1.**
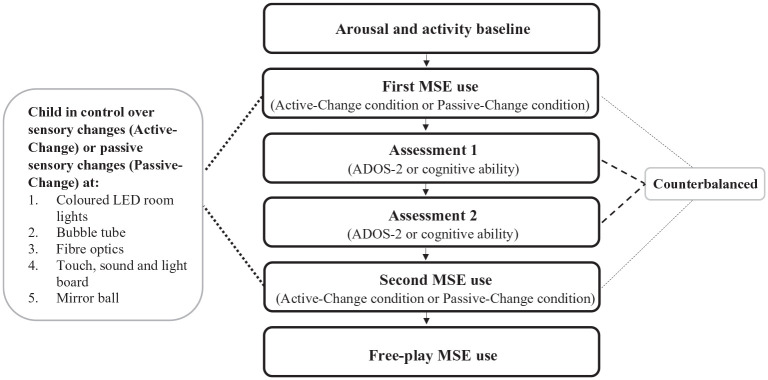
Procedure of each testing session. The Multi-Sensory Environment (MSE) conditions, Active-Change condition and Passive-Change condition, were counterbalanced, as were the Autism Diagnostic Observation Schedule (ADOS-2) and cognitive ability assessments (e.g. Wechsler Abbreviated Scale of Intelligence).

**Figure 2.**
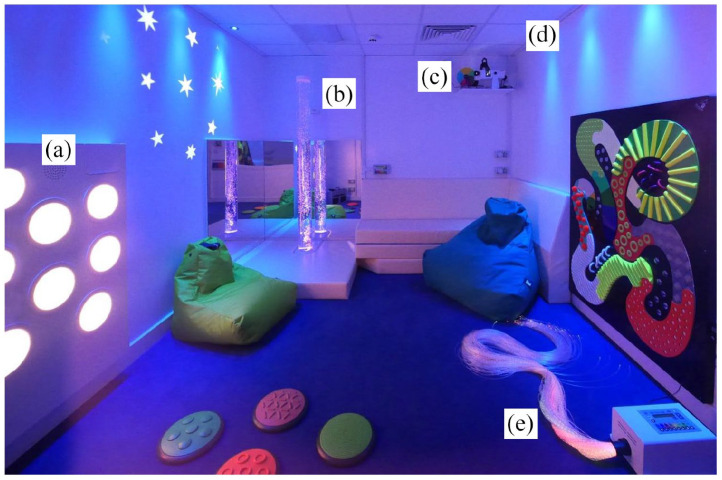
Image of the Multi-Sensory Environment used in this study containing, (a) touch, sound and light board, (b) bubble tube, (c) pin spot for adjacent mirror ball (not in frame), (d) coloured LED room lights and (e) fibre optics. The projected wall stars and textured floor dots were not included for the MSE sessions in this study (photo courtesy of Mike Ayres).

Participants used the MSE with an experimenter in two different conditions, an Active-Change condition and a Passive-Change condition. The procedure of each testing session is outlined in Figure 1 and the MSE used in the study is shown in Figure 2. Detailed descriptions of the MSE equipment used in the study, and how they were used in each condition, can be found in Table 2.

**Table 2.** Equipment within the Multi-Sensory Environment (MSE) used in the study and how it can be engaged with in each condition.

Item and description	Active-Change condition	Passive-Change condition
(a) Coloured LED room lights: lights positioned on the ceiling that change the colour of the room	One of the eight colours can be selected, changing the colour as often as they choose	One of the eight alternating colours is presented every 3 s
(b) Bubble tube: water-filled tube with bubbles that travel up the tube continuously	One of the eight colours can be selected, changing the colour as often as they choose	One of the eight alternating colours is presented every 3 s
(c) Fibre optics: fibre optic cabling that lights up	One of the eight colours can be selected, changing the colour as often as they choose	One of the eight alternating colours is presented every 3 s
(d) Touch, sound and light board: board with eight buttons; when touched they produce a coloured light and a sound	Eight buttons can be pressed on the equipment lighting up one of the eight colours and playing a piano note. Buttons can be pressed concurrently	Eight buttons, each of a different colour, are lit. Each colour changes position on the board every 3 s. No piano notes are played
(e) Mirror ball: pin-spot light shines on a spinning mirror ball, projecting small moving lights onto the walls	Activate and de-activate the spotlight that shines onto the continually rotating mirror ball	The spotlight remains active throughout

Each participant spent 3 min with each of the five pieces of equipment in both MSE conditions, totalling 15 min per condition. The order of equipment use was determined using a Latin square, with the same equipment order used across both conditions by each participant. The order of the MSE conditions was counterbalanced across participants. In both conditions, each piece of equipment was only activated during the allocated 3-min visit, and the iPad was only available during the Active-Change condition. Both sessions were recorded using three wall-mounted cameras, which captured the whole of the 12.44 m^2^ space. Two cameras were placed in top-corners, angled down to the opposite side of the room, and one camera was placed in the top-centre of a wall opposite the other cameras, facing down across the space.

Due to a technical fault, the mirror ball was not available in one condition for five participants. For these participants, the mirror ball was excluded from analyses in both conditions and the overall means were adjusted accordingly.

The session started with an arousal and activity baseline, which is described below, and ended with a 5-min free play session that did not form part of this study. In the Active-Change condition, the participants changed sensory aspects of the equipment themselves using an iPad, or by directly pressing the equipment when at the touch, sound and light board (TSLB). Activation of the equipment using the iPad was achieved with S4 software (Mike Ayres Design, 2012). In the Passive-Change condition, each respective piece of equipment, apart from the mirror ball, changed colour every 3 s without the participant’s input. The mirror ball was activated and rotated throughout the 3 min (see Table 2).

To control for experimenter speech across conditions, standard instructions were devised. In the Active-Change condition, the relevant piece of equipment was activated and the iPad was handed to the child with the instruction, ‘You can play’. At the TSLB, the experimenter used the same instructions while gesturing towards the board. If the child did not press a button, one press was demonstrated by the experimenter with the instruction, ‘Look, like this’. Most participants used the iPad or TSLB after the first instruction. In the Passive-Change condition, the instruction, ‘You can play’ was provided without the iPad or gesture. If the participant moved away from the equipment during the 3 min in either condition, specific phrases were used to orient them back: ‘Not finished yet’, ‘Play over here’ and ‘You can play’. In addition, four utterances were said during each equipment use, which provided explicit opportunities for the participant to engage with the experimenter. Two of the four utterances were factual statements about the piece of equipment (e.g. ‘Look, that red is bright’), while the other two were imaginative (e.g. at the fibre optics, ‘Look, it’s like wiggly worms’). One of each type of statement was combined with an action to provide an opportunity for mimicry (e.g. ‘Look, it’s like a jelly fish’, accompanied by waving the fibre optic cables like tentacles). Apart from these utterances and the instructions, the experimenter only spoke if the participant asked a question and responses were kept to a minimum.

To measure participant HRV and activity, a chest monitor (Actiwave Cardio, CamNTech, 2018b) was worn. Before the MSE sessions, a baseline measure of arousal was taken where the participant played independently with a range of age-appropriate toys for 5 min in a standard testing room, these included a marble run game, building bricks, toy cars and a puzzle. None of the toys had explicit sensory qualities (e.g. lights). The experimenter did not engage with the participant during this session unless the participant asked a question. The parent was not present during any of the MSE or baseline sessions.

#### Outcome measures

In our previous qualitative study, practitioners reported the behavioural and cognitive changes that they regularly observed in autistic children when they were using an MSE (Unwin et al., 2021). We used this data to develop the outcomes for this study, which included core autistic features: RMBs, sensory behaviour, and social interaction and communication (including social behaviour, gestures, mimicry, speech and rapport) and other behaviours: attention, anxiety and positive affect.

The coding scheme was devised using best practice guidelines (Boateng et al., 2018), beginning with domain identification and item generation through deductive methods (see Table 1 in Boateng et al., 2018). Following this, items were evaluated by the research team and an expert in behavioural coding external to the team, and were deemed relevant and representative of the variables. The scheme was then used to code trial videos to assess its utility. During this phase, inductive item development was used to refine definitions based on observed behaviours. Test–retest reliability could not be assessed as it was not feasible to ask participants to come into the centre more than once. However, the coding scheme had close adherence with previously validated measures (discussed below), with our primary intention to adapt them to be fit for purpose for our experiment. The coding scheme is available upon request.

Variables were generally measured using frequency (number of discrete events) and/or duration (length of event, in seconds), as appropriate. Behaviours were coded for each piece of equipment (Table 3), unless otherwise specified, and summed across equipment to create an overall score.

**Table 3.** Coding categories and behaviours used to measure behaviour across conditions.

Coding category	Code	Subcode	Coding type
Repetitive motor behaviours	Whole body		Frequency and duration
	Hand/finger/foot		
	Locomotion		
Sensory	Defensive visual		Frequency and duration
	Defensive auditory		
	Defensive tactile		
	Seeking visual		
	Seeking auditory		
	Seeking tactile		
Social communication	Speech	Speech (any that is not stereotyped/idiosyncratic)	Frequency
		Stereotyped/idiosyncratic	
		Vocalisations	
	Social behaviour	Showing	
		Requesting	
		Offering information	
		Asks for information	
		Shared enjoyment	
	
	Gestures	Conventional	
		Informational	
		Emphatic/beats	
		Deictic	
	Mimicry	Full mimic	
		Partial mimic	
	
	Rapport		Global rating
Anxiety	Whine or whimper		Frequency
	Stutter		
	Trembling/shaking body or voice		
	Jumpiness		
	Body contortions or rigid posture		
	Physical complaint		
	Desire to leave		
	Expression of fear or worry		
	Cry		
	Irritable		
Positive affect	Smiling		Frequency
	Laughing		
	Verbal expressions of enjoyment		
	Integrated singing and dancing		
Attention			Global rating

##### RMBs

This measure included items adapted from previous MSE studies (J. Chan & Chien, 2017; S. Chan et al., 2005; Shapiro et al., 1997) and RRB questionnaires (Repetitive Behaviour Scale–Revised, RBS-R, Bodfish et al., 2000; Repetitive Behaviour Questionnaire-2, RBQ-2, Leekam, Tandos, et al., 2007). The final RMB measure included three behavioural categories: repetitive whole-body movements, repetitive hand/finger/foot movements and repetitive locomotive movements, which were combined to create a total score. Frequency and duration were measured.

##### Sensory behaviours

This measure drew upon validated sensory observation measures (Sensory Processing Assessment, SPA, Baranek, 1999b; Sensory Assessment for Neurodevelopmental Disorders, SAND, Siper et al., 2017), the ADOS-2 (Lord et al., 2012) and questionnaires (Sensory Experiences Questionnaire, SEQ, Baranek, 1999a; Sensory Profile, SP, Dunn, 1999; Glasgow Sensory Questionnaire, GSQ, Robertson & Simmons, 2013). The final scheme included measurement of specific behaviours categorised as sensory seeking or sensory defensive behaviours in the auditory, visual and tactile domains. Domains were combined for analysis to create three outcome measures: sensory seeking behaviours, sensory defensive behaviours and total sensory behaviours (combining seeking and defensive behaviours). Frequency and duration were measured.

##### Social interaction and communication

This was composed of five measures: social behaviour, gestures, mimicry, speech and rapport. Each measure was adapted from coding schemes in previous MSE studies (e.g. J. Chan & Chien, 2017) and the ADOS-2 (Lord et al., 2012). The social behaviour measure included five items: showing, requesting, offering information, asks for information and shared enjoyment. The gesture measure included four items: conventional, informational, emphatic and deictic. Mimicry included two items: full mimicry and partial mimicry. The frequency of these behaviours was coded, and a total score was calculated for each measure by summing the individual items. Both frequency and duration were coded for the speech measure, which included three items – speech, stereotyped/idiosyncratic speech (e.g. echolalia and reciting non-personal facts) and vocalisations. There was no total score for speech because of variation in the direction of the effects, with increased speech indicating more typical communication but the opposite being true for stereotyped/idiosyncratic speech. The rapport measure was adapted from ADOS-2 global coding, which included four levels of rapport (0–3), with a higher score indicating more atypical rapport and, therefore, a less comfortable interaction overall between child and experimenter. Rapport was rated across the whole condition, rather than at each piece of equipment.

##### Anxiety

This measure was informed by the Preschool Observation Scale of Anxiety (POSA; Glennon & Weisz, 1978), the Screen for Child Anxiety Related Disorders (SCARED; Birmaher et al., 1999), Spence’s Children’s Anxiety Scale (SCAS; Spence, 1999), Paediatric Anxiety Rating Scale (PARS; Riddle, 2002) and Anxiety Scale for Children-Autism Spectrum Disorder (ASC-ASD; Rodgers et al., 2016). The final items were: whine/whimper, stutter, trembling/shaking body or voice, jumpiness, body contortions/rigid posture, physical complaint, desire to leave, verbal expression of fear or worry, crying and irritability. The frequency of each behaviour was recorded, and variables were summed to create a total anxiety score.

##### Positive affect

Previous research describing the behavioural presentation of positive affect was used to create the positive affect measure (e.g. Harter, 1974), with the final measure comprising of four codes: smiling, laughing, verbal expressions of enjoyment, and integrated singing and dancing. Following advice from an expert in behavioural coding, a smile was only coded when the whole of the child’s face was visible. As the child’s face was visible in one camera angle most of the time, most smiles were captured. There was no minimum length for coding a smile. When laughing was coded, a smile could not be concurrently coded. Frequency of behaviours was coded, with the individual codes summed to create a total positive affect score.

##### Attention

This was a global rating that was informed by ADOS-2 (Lord et al., 2012), previous MSE studies (e.g. Cuvo et al., 2001), and broader literature on the conceptualisation of attention (e.g. Peterson et al., 1984). The measure rated the level of on-, and off-task behaviour on a scale of 0–2, with a higher score indicating more off-task behaviours (i.e. lower attention). An attention rating was assigned at the bubble tube, fibre optics, and TSLB but not at the room lights or mirror ball as these equipment affected sensory change in the whole MSE, which meant on- and off-task behaviour could not be reliably determined.

### Coding procedure

The videos, with three camera angles for each video, were coded by a primary and secondary coder. The secondary coder, naïve to the study hypotheses, double coded a random selection of 25% of the videos for calculation of IRR. The secondary coder was trained by the primary coder through: (1) discussions of the coding scheme, (2) observation and discussion of sample videos and (3) practice coding of sample videos (N.B. none of the sample videos were included in IRR coding). The coding was administered through viewing each video four times, with a systematic focus on different elements of the coding scheme each time the video was watched: (1) RMB and sensory, (2) social, (3) anxiety and positive affect and (4) attention. For each viewing of the video, continuous coding took place for the 3 min the child spent at each piece of equipment, resulting in 15 min of coding per condition. Note the coding period was manually constrained to 3 min by the primary coder.

Intraclass correlations were calculated on the 25% of videos that were double coded and these analyses demonstrated excellent IRR for all coding schemes (intraclass correlation coefficient (ICC) = 0.84–0.99) apart from sensory duration, which had moderate but acceptable reliability (ICC = 0.55; Cicchetti, 1994; Koo & Li, 2016). Although the coders agreed on the frequency sensory behaviours (ICC = 0.89), the second coder misread one item of the duration coding causing one sensory behaviour to be coded longer, and another to be coded shorter than the scheme dictated. Removing this one misunderstanding significantly improved IRR (ICC = 0.81).

To fully understand the data, frequency and duration of experimenter speech, number of iPad presses as an index of the number of sensory changes in the Active-Change condition and duration of iPad holding in the Active-Change condition were also measured. We also checked for procedural fidelity using an additional independent coder (blind to study hypotheses). The coder assessed: (1) that the five pieces of equipment were visited in each condition, (2) that the pieces of equipment were visited for 3 min each and (3) that the experimenter spoke the four prescribed utterances at each piece of equipment. The coder looked at the procedural fidelity for just over 25% of participants, who were selected at random (*n* = 11). Procedural fidelity was high across all three areas of assessment. The five pieces of equipment were visited for all participants, although the mirror ball failed to work for two participants (91% overall fidelity). For the length of time spent at the equipment, 95% of sessions were within ±10% of 3 min. Finally, there was an accuracy rate of 97% in delivering the four utterances at each piece of equipment.

To assess coding drift (i.e. change in coding over time), the first coded participants (*n* = 15) were compared to last coded participants (*n* = 15), across both conditions, as well as across all variables. There were no significant differences in participant codes over time (range of values *t* = 0.18–1.83, *p* = 0.08–0.98).

### Data preparation and analysis

Videos of the sessions were coded using ELAN software (ELAN, 2018) and then exported into SPSS (IBM Corp., 2017) for analysis.

Actiheart software (version 5.0.5., CamNTech, 2018a was used to extract and clean the HRV data and to convert the activity data into ‘activity counts’. HRV was analysed as a measure of arousal using the root mean square successive difference (RMSSD; see Berntson et al., 2005).

Variables were inspected for normality of distribution through visual inspection (e.g. histograms and box plots; degree of conformation to bell-curve form), the Shapiro–Wilk test, and skewness and kurtosis. Transformations were attempted for non-normal variables (frequency and duration of stereotypical speech, duration of RMB and sensory behaviours, and duration of whole-body and locomotive RMBs) and transformation type (square root and logarithmic) depended on the severity of the skewness of the data. Where the transformations did not improve the distribution (frequency and duration of stereotypical speech, and duration of whole-body and locomotive RMBs), non-parametric tests were used, and medians reported. For normally distributed data, paired-samples *t*-tests were used.

Where findings were significant, post hoc analyses investigated the difference in individual items. Given the number of comparisons across different domains of cognition and behaviour, we took a conservative approach of a significance threshold of *p* = 0.01 for the observation measures.

## Results

### Outcome variables

**Figure 3.**
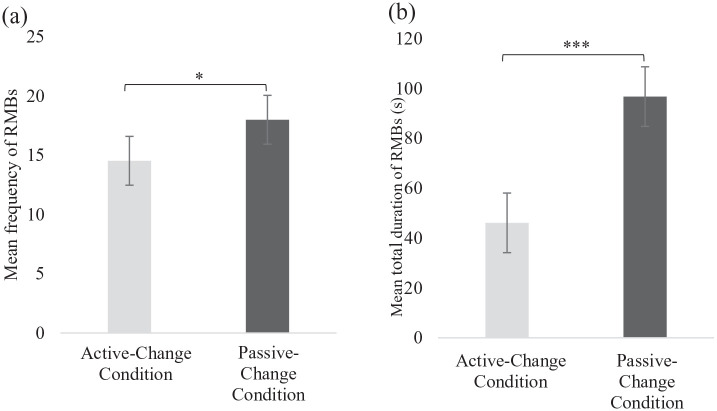
The (a) mean frequency and (b) mean total duration (in seconds) of Repetitive Motor Behaviours (RMBs) between the two conditions. Error bars represent the standard errors. *p < 0.05; ***p < 0.001.

RMBs were both fewer (*t*(40) = −2.08, *p* = 0.04, *d_z_* = −0.32) and shorter in duration (*t*(40) = 18.65, *p* < 0.001, *d_z_* = 2.91) in the Active-Change condition, compared to the Passive-Change condition (Figure 3).

Post hoc analyses showed fewer (*t*(40) = 16.16, *p* < 0.001, *d_z_* = 2.52) and shorter (*Z* = −1.98, *p* < 0.05, *r* = −0.31) repetitive whole-body movements in the Active-Change condition. The duration of repetitive hand/finger/foot (*t*(40) = −2.47, *p* < 0.05, *d_z_* = −0.39) and locomotion (*Z* = 3.27, *p* < 0.001, *r* = 0.51) movements were also significantly shorter in the Active-Change condition.

**Figure 4.**
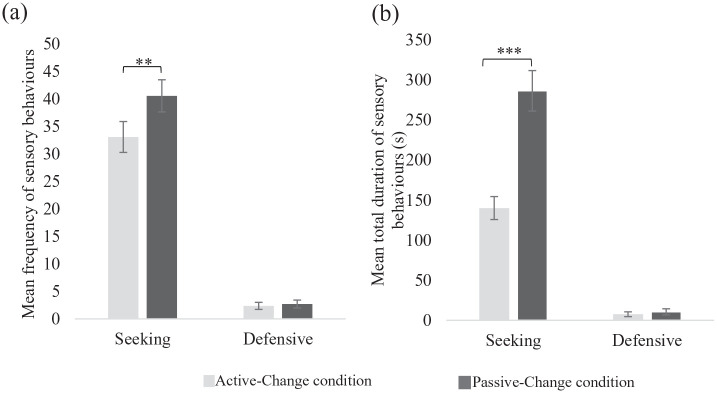
The (a) mean frequency and (b) mean total duration of seeking and defensive behaviours across conditions. Error bars represent the standard error. **p < 0.01; ***p < 0.001.

There were significantly fewer sensory behaviours in the Active-Change condition (M = 35.46, SD = 17.80) compared to the Passive-Change condition (M = 43.29, SD = 18.63), *t*(40) = −2.82, *p* = 0.007, *d_z_* = −0.44. Sensory behaviours were also shorter in the Active-Change (M = 147.87, SD = 94.53) compared to the Passive-Change condition (M = 297.29, SD = 167.30), *t*(40) = 15.53, *p* < 0.001, *d_z_* = 2.43. Post hoc analyses (Figure 4) showed that there were fewer (*t*(40) = −2.75, *p* < 0.01, *d_z_* = −0.43) and shorter lasting (*t*(40) = 16.22, *p* < 0.001, *d_z_* = 2.53) seeking behaviours in the Active-Change, compared to the Passive-Change condition. In contrast, there were very few defensive behaviours in either condition and no difference in the frequency (*t*(40) = −0.92, *p* = 0.36, *d_z_* = −0.14) or duration (*t*(40) = −0.32, *p* = 0.19, *d_z_* = −0.05) of these behaviours.

**Figure 5.**
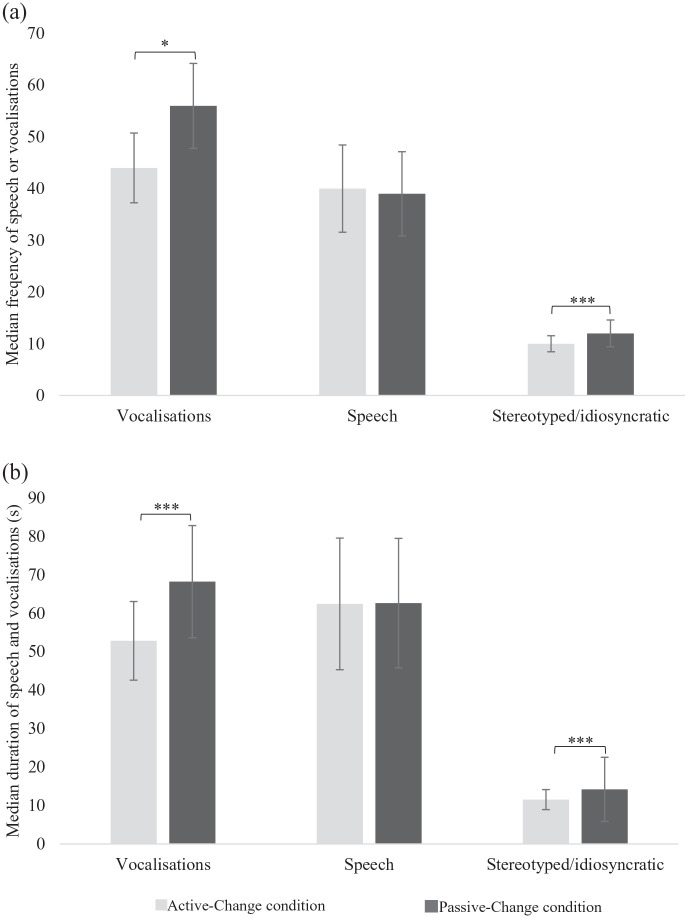
The median (a) frequency and (b) duration of vocalisations, speech (not including stereotyped/idiosyncratic) and stereotyped/idiosyncratic speech between conditions. Error bars represent the standard errors. *p < 0.05; ***p < 0.001.

Significantly more attention was paid in the Active-Change condition (M = 1.27, SD = 1.43) compared to the Passive-Change condition (M = 1.88, SD = 1.45), *t*(40) = −3.73, *p* < 0.001, *d_z_* = −0.58. There were fewer (*t*(40) = −10.66, *p* = 0.02, *d_z_* = −1.66) and shorter (*t*(40) = 20.79, *p* < 0.001, *d_z_* = 3.25) vocalisations in the Active-Change condition. Along with less (*Z* = 5.51, *p* < 0.001, *r* = 0.86) and shorter (*Z* = 5.51, *p* < 0.001, *r* = 0.86) stereotyped/idiosyncratic speech in the Active-Change condition. However, there were no significant differences for speech (both *p* > 0.05) (Figure 5).

There was no significant difference in the frequency of the other variables between conditions (see Table 4).

**Table 4.** Mean values, standard deviations (SD; in brackets), *t*-scores, *p*-values and effect sizes (*d_z_*) of the number of social behaviours, gestures, mimicry, anxiety and positive affect behaviours in the Active-Change or Passive-Change conditions. Rapport is a global rating score (range = 0–3).

	Active-Change Mean (SD)	Passive-Change Mean (SD)	*t*-score	*p*-value	*d_z_*
Social behaviours	15.68 (19.77)	16.54 (23.55)	−0.66	0.52	−0.10
Gestures	4.95 (6.28)	6.54 (8.26)	−1.78	0.08	−0.28
Mimicry	2.10 (1.77)	2.63 (1.88)	−1.73	0.09	−0.27
Rapport	1.73 (0.95)	1.78 (1.08)	−0.53	0.60	−0.08
Anxiety	5.73 (6.00)	6.61 (6.32)	−1.24	0.22	−0.19
Positive affect	16.83 (15.71)	14.32 (11.65)	1.65	0.11	−0.26

### Arousal

**Figure 6.**
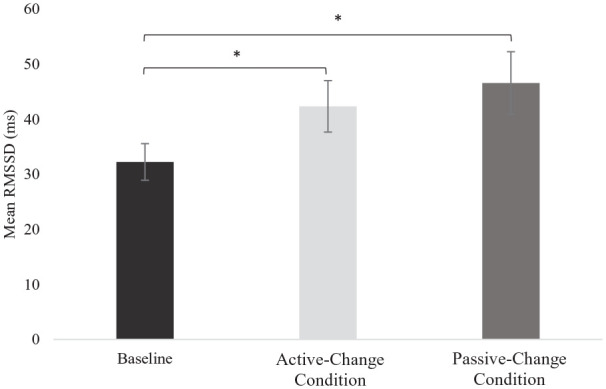
Mean heart rate variability (root mean square of the successive differences, RMSSD) across the baseline, Active-Change and Passive-Change conditions. Error bars represent the standard error. Higher HRV indicates the low arousal. *p < 0.05.

There was a main effect of condition on HRV (RMSSD; *F*(2, 38) = 3.70, *p* = 0.03, *η*^2^ = 0.16; Figure 6). Post hoc tests revealed significantly lower RMSSD (higher arousal) in the baseline condition compared to both the Active-Change (*t*(19) = −2.10, *p* < 0.05, *d_z_* = −0.47) and Passive-Change (*t*(19) = −2.45, *p* < 0.05, *d_z_* = −0.55) conditions. However, there was no significant difference between the Active-Change and Passive-Change conditions, *t*(19) = −0.77, *p* = 0.45, *d_z_* = −0.17.

**Figure 7.**
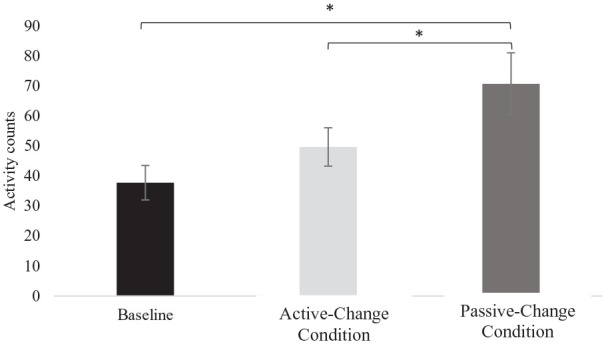
Activity level (activity counts from accelerometery data) across the baseline, Active-Change and Passive-Change conditions. Error bars represent the standard error. *p < 0.05.

A repeated measures analysis of variance (ANOVA) showed a main effect of condition on activity levels (*F*(2, 38) = 5.41, *p* < 0.01, *η*^2^ = 0.22). Post hoc tests established less activity in both the Active-Change (*t*(19) = −2.15, *p* < 0.05, *d_z_* = 0.48) and baseline conditions (*t*(19) = −2.86, *p* = 0.01, *d_z_* = −0.64) compared to the Passive-Change condition. However, baseline activity was not significantly different to activity in the Active-Change condition (*t*(19) = −1.33, *p* > 0.05, *d_z_* = −0.30; Figure 7).

### Use of the iPad and TSLB in the Active-Change condition

The number of presses on the iPad and TSLB was used as a measure of the frequency of sensory changes in the Active-Change condition. In the Passive-Change condition, each piece of equipment was automatically set to change every 3 s except for the mirror ball, which was continuously active. There were more changes to the sensory environment in the Active-Change (M = 364.93, SD = 243.98) compared to the Passive-Change (M = 240, SD = 0) condition (*t*(40) = 3.28, *p* = 0.002, *d_z_* = 0.51).

The iPad was held for an average total duration of 103 s (SD = 121), across the Active-Change condition. Spearman’s Rho correlations investigated whether the degree of iPad and TSLB presses and duration of iPad holding in the Active-Change condition were associated with a reduction in behaviours involving the hands (i.e. RMBs, sensory behaviours, gestures and mimics). Correlations were small with only one, in the opposite direction than expected, reaching significance, as the longer the iPad was held, the more gestures were produced (*r_s_* = 0.40, *p* = 0.009; other variables *r_s_* = −0.36 to 0.02, n.s.). Correlations with iPad and TSLB presses were not significant (*r_s_* = −0.20 to 0.39).

### Experimenter speech

There was no significant difference in the frequency (M_difference_ = 1.78, SD = 18.73; *t*(40) = 0.61, *p* = 0.55, *d_z_* = 0.10) or duration (M_difference_ = −0.03, SD = 21.63; *t*(40) = −0.01, *p* = 0.99, *d_z_* = 0.001) of experimenter speech between the two conditions.

## Discussion

We investigated the effect of having control over sensory equipment on the behaviours of autistic children using an MSE. We found that having control led to a significant reduction in RMBs, sensory behaviours, activity, stereotyped speech and vocalisations, and an increase in attention. However, we found no significant changes in social behaviours, anxiety, positive affect or arousal. The changes, which we argue provide better conditions for learning, resonate with reports of autistic preference for being in control (Ashburner et al., 2013; Fujino et al., 2019; Robertson & Simmons, 2015), along with perceptual (Palmer et al., 2017; Pellicano & Burr, 2012; Powell et al., 2016; Van De Cruys et al., 2014) and cognitive (Boulter et al., 2014; Wigham et al., 2015) accounts that position predictability and control as important determinants of autistic experience.

### Why does being in control affect outcomes?

The importance of control is becoming an increasingly recognised in autism, and to our knowledge, this is the first study to directly assess the impact of providing control on a range of outcomes in an MSE. We have three interpretations for why control may have changed outcomes: meeting sensory needs, predictability and agency/choice.

First, giving children control of the sensory equipment in the room could ensure that they are able to meet their sensory needs. Previous research, both in an MSE (Mey et al., 2015) and more broadly (e.g. Lane et al., 2010), has suggested that meeting sensory needs can result in a range of positive outcomes for autistic children. MSEs are a potentially important tool in meeting sensory needs as they offer a wide range of sensory experiences, necessary when considering the complex sensory heterogeneity of autism (e.g. Lane et al., 2010).

Second, being in control may have increased the predictability of the sensory environment. Predictability is at the core of many leading accounts of sensory processing in autism, such as Bayesian and predictive coding theories (e.g. Pellicano & Burr, 2012; Van De Cruys et al., 2014). Broadly, these theories suggest that perceptual experience in autistic individuals is driven relatively more by bottom-up sensory information than top-down predictions based on prior experience. This renders the sensory world more surprising and could lead to increases in factors such as anxiety and cognitive load (Van De Cruys et al., 2014). In this study, giving the children control of the sensory equipment might have enabled them to better predict their own sensory experience and reduce feelings of ‘sensory overload’. This could explain why we found a reduction in RMBs and an increase in attention, as both have previously been related to a need for control and predictability (Boulter et al., 2014; Kapp et al., 2019; Pellicano & Burr, 2012). For example, an account from an autistic adult described how ‘the regulatory aspect of stimming worked through attending to a single point of focus over which one had control’ (Kapp et al., 2019, p. 1785). It is important to acknowledge that we did not manipulate social predictability or control in this study, which may explain why social behaviours were unchanged across conditions.

Our third hypothesis for why control of sensory changes produced positive outcomes is based on the effects of increased agency and choice, which are strongly related to well-being in children (Fattore et al., 2007; Fattore et al., 2009). Research has suggested that the perception of being in control of the sensory environment can be just as important as the sensory changes that are actually made (Pfeiffer et al., 2017). Therefore, it is possible that some of the positive outcomes we observed in this study were not due to the sensory changes per se, but due to the positive effects of increased agency and choice over the equipment in the room.

We did not have the scope to explore an exact mechanism for how control over sensory changes within an MSE could change behaviour. It is worth noting that although the participants in the Active-Change condition had control of the piece of equipment they were working with, they did not have *full* control of their experience within the MSE as equipment order and the duration of each activity was predetermined. If agency and choice are driving the findings, then the more autonomy autistic children have over decisions in the MSE, the bigger the cognitive and behavioural effects. In contrast, sensory predictability would not be improved by increasing the amount of decision-making. In terms of the meeting of sensory needs, the effect of increasing the number of decisions or elements to control would depend on the specific sensory needs of the child (e.g. a hypersensitive child would not benefit from being able to choose to turn all equipment on). Therefore, there is scope to tease apart these mechanisms with experimental manipulations. Having said this, we do not assume these theories to be mutually exclusive or exhaustive. Ideas around controlling the sensory environment, and therefore sensory experience, are consistent with existing theories around predictability and perceived agency and choice in autism. These ideas also align with other interventions that enable autistic individuals to control their sensory environments, such as wearing headphones (Ikuta et al., 2016; Pfeiffer et al., 2019; Rowe et al., 2011) and modification of lighting (e.g. Kinnealey et al., 2012).

### Relevance for learning

The potential of MSEs to support learning has been reflected in a range of MSE commentaries (Fowler, 2008; Hirstwood & Smith, 1996), as well as qualitative (Ayer, 1998; Stephenson & Carter, 2011) and quantitative studies (Houghton et al., 1998; Mey et al., 2015). Learning in MSEs has been described in relation to specific skills such as understanding cause-and-effect (Fowler, 2008; Hirstwood & Smith, 1996), where a user activating the equipment can learn that an action (e.g. pressing a switch) can effect change (e.g. the bubble tube activates). Being in control of the sensory changes is likely to increase this understanding of causal learning. However, it is also possible that being in control over the sensory changes in the room could have an indirect impact on learning via interactions with other behaviours.

In a previous study, we found that practitioners working with autistic children are aware that control is an important component of MSE use, and they thought it could influence learning outcomes in a number of different ways (Unwin et al., 2021). An advantage of this study is that it looked beyond core autistic features to behaviours practitioners described as relevant for autistic children using MSEs (Unwin et al., 2021). Many of the behaviours that we found were influenced by being in control are also related to learning outcomes, for example, RRBs (Leekam et al., 2011), sensory behaviours (Jones et al., 2020), and activity levels and attention (Reichenbach et al., 1992). It has also been suggested that 30% of school teachers of autistic children think that sensory differences affect learning *all* of the time (Jones et al., 2020). Therefore, it could be hypothesised that providing control may improve conditions for learning, with further research required to examine the mechanisms of this effect. A notable consideration is that we investigated children working 1:1 with an adult in the MSE, where control of the sensory environment is easy to achieve. However, MSE sessions are often delivered for small groups of children. Taking turns to be in control of the MSE or providing control to each child at different pieces of equipment are ways in which the experience of sensory autonomy could be supported during group sessions. The extent to which these adaptations replicate the behavioural effects of a 1:1 session is an area of investigation that could be of particular benefit to practitioners.

### Consideration of neurodiversity

It is important to note that although being in an environment that enables behaviour change may suggest a more comfortable experience, improving or reducing particular outcomes (e.g. RMBs) would not necessarily be beneficial or wanted by all autistic people. For example, RRBs have been associated with enjoyment (Joyce et al., 2017) and autistic people have described liking their RMBs, if not the social consequences that accompany them (Kapp et al., 2019). In line with this, the neurodiversity movement argues for an acceptance of autistic behaviours and a rejection of approaches that may seek to ‘cure’ autism (Kapp et al., 2013).

We do not suggest that it is a positive outcome of MSE use to reduce or eliminate autistic behaviours per se. Rather, we seek to find methods of use that best support opportunities for the development and learning of autistic children. Where autistic behaviours may indicate discomfort or interfere with learning, then their reduction may lead to positive benefits for the child.

### Behaviours unaffected by control in the MSE

Although control over the sensory changes in the MSE resulted in changes to some outcomes, others such as social communication, positive affect, anxiety and arousal were unaffected. This could suggest that these outcomes are not affected by having control, but it could also reflect the ‘dose’ of intervention needed or challenges of measurement. For example, opportunities for social interaction were limited in this study because engagement with the experimenter was controlled. Two previous MSE studies on intellectually disabled children found that increasing practitioner/caregiver interaction increased social communication, but only when interaction from the other person was high (Glenn et al., 1996; Hill et al., 2012). Relatedly, Mey et al. (2015) found an improvement in social communication in their six autistic participants but only after a year of MSE intervention. Furthermore, we may not have been able to capture the full emotional experience of our participants, because their emotions may not have been expressed visibly or they may have been expressed in a way that was not obvious or typical to the coder (Keating & Cook, 2021).

These measurement considerations reflect the challenges of observational data, especially in an inclusive and heterogeneous group of participants. However, generally our observational coding scheme worked well and was able to capture many of the different behaviours and characteristics associated with autism. Future research should examine the subjective experience of children using the MSEs. This was beyond the scope of this study but is crucial to fully understand how different ways of using the MSE can impact behaviour, affect and learning.

### Limitations

The commercial MSE equipment used, although important for the ecological validity of the study, did not allow us to match the rate of sensory changes in the Active-Change condition to the Passive-Change condition. Although it is also worth noting that a matched design would have introduced order effects between the conditions. However, this also meant that there were more sensory changes in the Active-Change condition, and we cannot discount that this underlying discrepancy had an impact on the findings. An additional difference between conditions was that the iPad was only available in the Active-Change condition, where it was the mechanism of control for four of the pieces of equipment. It is not possible to discount that some of the difference between conditions could be related to the presence or absence of the iPad, rather than the child being in control. Although future work should endeavour to control for the number of changes and the inclusion of equipment, the findings are still clear in demonstrating that giving an autistic child control of the MSE leads to behaviour change, regardless of the mechanisms of action. This study cannot provide insight into the benefit of MSE use over engagement in a comparable but non-sensory environment (e.g. regular playroom), and indeed, there was significantly higher arousal in the baseline (i.e. 5 min free play outside the MSE) compared to the two MSE conditions. However, as the baseline condition was always the first activity, the difference in arousal could reflect order effects. More broadly, there are challenges in choosing an appropriate non-MSE condition as an environment with reduced sensory interest and stimulation is a poor control for the natural interest provided by an MSE. Relatedly, our findings may not be unique to MSEs; being in control could potentially have an impact across other contexts (e.g. classrooms). Therefore, future research could further investigate the impact of providing control over the sensory environment in a non-MSE setting.

With reference to specific variables, the static camera system did not allow for close viewing of the child’s face, limiting our ability to code affect using a fine-grained coding system such as the Facial Action Coding System (e.g. Sayette et al., 2001). This may have limited our ability to detect changes in affect between conditions. We would encourage those undertaking future research to utilise moving camera systems, capable of zooming in and out, to support this type of data collection. With relation to speech, we found that vocalisations were reduced when the child had control of the MSE; however, the intent of vocalisations (communicative intent vs sensory stimulation) could not be reliably determined. Our tentative interpretation is that most were sensory seeking, suggesting that the findings are consistent with the overall reduction in sensory seeking in the Active-Change condition. An additional consideration is that coders were not naïve to condition. However, the second coder was blind to hypotheses and inter-rater reliability (IRR) was generally excellent across the coding scheme, which supports the validity of our data. Finally, as data were not collected on ethnicity, race or socioeconomic status, we cannot infer the extent to which the results generalise across different populations.

## Conclusion

This is the largest study to date on MSE use with autistic children and the first to investigate the impact of having control over sensory changes within an MSE. Compared to not having control, we found that giving autistic children control of the MSE reduced their RMBs, sensory behaviours, activity levels, stereotyped speech and vocalisations, and increased their attention. These findings speak to a broader literature that has identified the importance of control for autistic children. The data are also promising for practitioners who currently have no evidence-based guidelines to inform their MSE practice with autistic children.

## References

[bibr1-13623613211050176] American Psychiatric Association. (2013). Diagnostic and statistical manual of mental disorders (5th ed.). American Psychiatric Publishing.

[bibr2-13623613211050176] Ashburner J. Bennett L. Rodger S. Ziviani J. (2013). Understanding the sensory experiences of young people with autism spectrum disorder: A preliminary investigation. Australian Occupational Therapy Journal, 60(3), 171–180. 10.1111/1440-1630.12025 23730782

[bibr3-13623613211050176] Ayer S. (1998). Use of multi-sensory rooms for children with profound and multiple learning disabilities. Journal of Intellectual Disabilities, 2(2), 89–97. 10.1177/146900479800200206

[bibr4-13623613211050176] Baillon S. van Diepen E. Prettyman R. (2002). Multi-sensory therapy in psychiatric care. Advances in Psychiatric Treatment, 8(6), 444–450. 10.1192/apt.8.6.444

[bibr5-13623613211050176] Baranek G. (1999a). Sensory experiences questionnaire (version 2.1): Manual. University of North Carolina.

[bibr6-13623613211050176] Baranek G. (1999b). Sensory Processing Assessment for Young Children (SPA) [Unpublished manual, University of North Carolina at Chapel Hill].

[bibr7-13623613211050176] Berntson G. Lozano D. Chen Y. (2005). Filter properties of root mean square successive difference (RMSSD) for heart rate. Psychophysiology, 42(2), 246–252. 10.1111/j.1469-8986.2005.00277.x 15787862

[bibr8-13623613211050176] Berument S. Rutter M. Lord C. Pickles A. Bailey A. (1999). Autism screening questionnaire: Diagnostic validity. British Journal of Psychiatry, 175, 444–451. 10.1192/bjp.175.5.444 10789276

[bibr9-13623613211050176] Birmaher B. Brent D. A. Chiappetta L. Bridge J. Monga S. Baugher M. (1999). Psychometric properties of the Screen for Child Anxiety Related Emotional Disorders (SCARED): A replication study. Journal of the American Academy of Child and Adolescent Psychiatry, 38(10), 1230–1236. 10517055 10.1097/00004583-199910000-00011

[bibr10-13623613211050176] Boateng G. O. Neilands T. B. Frongillo E. A. Melgar-Quiñonez H. R. Young S. L. (2018). Best practices for developing and validating scales for health, social, and behavioral research: A primer. Frontiers in Public Health, 6, Article 149. 10.3389/fpubh.2018.00149 PMC6004510 29942800

[bibr11-13623613211050176] Bodfish J. Symons F. Parker D. Lewis M. (2000). Varieties of repetitive behavior in autism: Comparisons to mental retardation. Journal of Autism and Developmental Disorders, 30(3), 237–243. 10.1023/A:1005596502855 11055459

[bibr12-13623613211050176] Botts B. Hershfeldt P. Christensen-Sandfort R. (2008). Empirical review of product representation. Focus on Autism and Other Developmental Disabilities, 23(3), 138–147.

[bibr13-13623613211050176] Boulter C. Freeston M. South M. Rodgers J. (2014). Intolerance of uncertainty as a framework for understanding anxiety in children and adolescents with autism spectrum disorders. Journal of Autism and Developmental Disorders, 44(6), 1391–1402. 10.1007/s10803-013-2001-x 24272526

[bibr14-13623613211050176] Boyd B. A. Baranek G. T. Sideris J. Poe M. D. Watson L. R. Patten E. Miller H. (2010). Sensory features and repetitive behaviors in children with autism and developmental delays. Autism Res, 3(2), 78–87. 10.1002/aur.124 20437603 PMC3071028

[bibr15-13623613211050176] Bozic N. (1997). Constructing the room: Multi-sensory rooms in educational contexts. European Journal of Special Needs Education, 12(1), 54–70. 10.1080/0885625970120106

[bibr16-13623613211050176] Cameron A. Burns P. Garner A. Lau S. Dixon R. Pascoe C. Szafraniec M. (2019). Making sense of multi-sensory environments: A scoping review. International Journal of Disability, Development and Education, 67(6), 630–656. 10.1080/1034912X.2019.1634247

[bibr17-13623613211050176] CamNTech. (2018a). Actiheart software, version 5.0.5. [Software]. https://www.camntech.com/actiheart-software/

[bibr18-13623613211050176] CamNTech. (2018b). Actiwave Cardio, [Apparatus]. https://www.camntech.com/cardio/

[bibr19-13623613211050176] Chan J. Chien W. (2017). A randomised controlled trial on evaluation of the clinical efficacy of massage therapy in a multisensory environment for residents with severe and profound intellectual disabilities: A pilot study. Journal of Intellectual Disability Research, 61(6), 532–548. 10.1111/jir.12377 28387017

[bibr20-13623613211050176] Chan S. Fung M. Tong C. Thompson D. (2005). The clinical effectiveness of a multisensory therapy on clients with developmental disability. Research in Developmental Disabilities, 26(2), 131–142. 10.1016/j.ridd.2004.02.002 15590244

[bibr21-13623613211050176] Cicchetti D. (1994). Guidelines, criteria, and rules of thumb for evaluating normed and standardized assessment instruments in psychology. Psychological Assessment, 6(4), 284–290. https://www.psycholosphere.com/Guidelines…EvaluatingNormed&StandardizedAssessmentInstrumentsbyCicchetti.pdf

[bibr22-13623613211050176] Cuvo A. J. May M. Post T. (2001). Effects of living room, Snoezelen room, and outdoor activities on stereotypic behaviour and engagement by adults with profound mental retardation. Research in Developmental Disabilities, 22, 183–204. 11380058 10.1016/s0891-4222(01)00067-1

[bibr23-13623613211050176] Dunn W. (1999). Sensory profile user’s manual. The Psychological Corp.

[bibr24-13623613211050176] ELAN. (2018). ELAN: Max Planck Institute for Psycholinguistics. [Software]. https://archive.mpi.nl/tla/elan

[bibr25-13623613211050176] Fattore T. Mason J. Watson E. (2007). Children’s conceptualisations of their well-being. Social Indicators Research, 80, 5–29. 10.1007/s11205-006-9019-9

[bibr26-13623613211050176] Fattore T. Mason J. Watson E. Mason J. Watson E. (2009). When children are asked about their well-being: Towards a framework for guiding policy. Child Indicators Research, 2, 57–77. 10.1007/s12187-008-9025-3

[bibr27-13623613211050176] Fava L. Strauss K. (2010). Multi-sensory rooms: Comparing effects of the Snoezelen and the Stimulus Preference environment on the behavior of adults with profound mental retardation. Research in Developmental Disabilities, 31(1), 160–171. 10.1016/j.ridd.2009.08.006 19815373

[bibr28-13623613211050176] Fowler S. (2008). Multisensory rooms and environments: Controlled sensory experiences for people with profound and multiple disabilities. Jessica Kingsley.

[bibr29-13623613211050176] Fujino J. Tei S. Itahashi T. Aoki Y. Ohta H. Kubota M. . . . Takahashi H. (2019). Need for closure and cognitive flexibility in individuals with autism spectrum disorder: A preliminary study. Psychiatry Research, 271, 247–252. 10.1016/j.psychres.2018.11.057 30504060

[bibr30-13623613211050176] Glenn S. Cunningham C. Shorrock A. (1996). Social interaction in multi-sensory environments. In Bozic N. Murdoch H. (Eds.), Learning through interaction: Technology and children with multiple disabilities, (pp. 67–82). David Foulton.

[bibr31-13623613211050176] Glennon B. Weisz J. R. (1978). An observational approach to the assessment of anxiety in young children. Journal of Consulting and Clinical Psychology, 46(6), 1246–1257. 10.1037/0022-006X.46.6.1246 730875

[bibr32-13623613211050176] Harter S. (1974). Pleasure derived from challenge and the effects of receiving grades on children’s difficulty level choices. Society for Research in Child Development, 49(3), 661–669. 4143822

[bibr33-13623613211050176] Hill L. Trusler K. Furniss F. Lancioni G. (2012). Effects of multisensory environments on stereotyped behaviours assessed as maintained by automatic reinforcement. Journal of Applied Research in Intellectual Disabilities, 25(6), 509–521. 10.1111/j.1468-3148.2012.00697.x 23055285

[bibr34-13623613211050176] Hilton C. Graver K. LaVesser P. (2007). Relationship between social competence and sensory processing in children with high functioning autism spectrum disorders. Research in Autism Spectrum Disorders, 1(2), 164–173. 10.1016/j.rasd.2006.10.002

[bibr35-13623613211050176] Hirstwood R. Smith C. (1996). Developing competencies in multi-sensory rooms. In Bozic N. Murdoch H. (Eds.), Learning through interaction: Technology and children with multiple disabilities, (pp. 83–91). David Fulton.

[bibr36-13623613211050176] Houghton S. Douglas G. Brigg J. Langsford S. Powell L. West J. . . . Kellner R. (1998). An empirical evaluation of an interactive multi-sensory environment for children with disability. Journal of Intellectual & Developmental Disability, 23(4), 267–278. 10.1080/13668259800033761

[bibr37-13623613211050176] IBM Corp. (2017). SPSS statistics for Windows (Version 25). [Software]. https://www.ibm.com/au-en/products/spss-statistics

[bibr38-13623613211050176] Ikuta N. Iwanaga R. Tokunaga A. Nakane H. Tanaka K. Tanaka G. (2016). Effectiveness of earmuffs and noise-cancelling headphones for coping with hyper-reactivity to auditory stimuli in children with autism spectrum disorder: A preliminary study. Hong Kong Journal of Occupational Therapy, 28, 24–32. 10.1016/j.hkjot.2016.09.001 30186064 PMC6091992

[bibr39-13623613211050176] Jones E. Hanley M. Riby D. (2020). Distraction, distress and diversity: Exploring the impact of sensory processing differences on learning and school life for pupils with autism spectrum disorders. Research in Autism Spectrum Disorders, 72, 1–12. 10.1016/j.rasd.2020.101515

[bibr40-13623613211050176] Joyce C. Honey E. Leekam S. R. Barrett S. L. Rodgers J. (2017). Anxiety, intolerance of uncertainty and restricted and repetitive behaviour: Insights directly from young people with ASD. Journal of Autism and Developmental Disorders, 47, 3789–3802. 10.1007/s10803-017-3027-2 28238024

[bibr41-13623613211050176] Kapp S. Gillespie-Lynch K. Sherman L. Hutman T. (2013). Deficit, difference, or both? Autism and neurodiversity. Developmental Psychology, 49(1), 59–71. 22545843 10.1037/a0028353

[bibr42-13623613211050176] Kapp S. Steward R. Crane L. Elliott D. Elphick C. Pellicano E. Russell G. (2019). ‘People should be allowed to do what they like’: Autistic adults’ views and experiences of stimming. Autism, 23(7), 1782–1792. 10.1177/1362361319829628 30818970 PMC6728747

[bibr43-13623613211050176] Keating C. Cook J. (2021). Facial expression production and recognition in autism spectrum disorders. Psychiatric Clinics of North America, 44(1), 125–139. 33526234 10.1016/j.psc.2020.11.010

[bibr44-13623613211050176] Kinnealey M. Pfeiffer B. Miller J. Roan C. Shoener R. Ellner M. (2012). Effect of classroom modification on attention and engagement of students with autism or dyspraxia. American Journal of Occupational Therapy, 66(5), 511–519. 10.5014/ajot.2012.004010 22917117

[bibr45-13623613211050176] Koo T. Li M. (2016). Cracking the code: Providing insight into the fundamentals of research and evidence-based practice a guideline of selecting and reporting intraclass correlation coefficients for reliability research. Journal of Chiropractic Medicine, 15, 155–163. 10.1016/j.jcm.2016.02.012 27330520 PMC4913118

[bibr46-13623613211050176] Lane A. Young R. Baker A. Angley M. (2010). Sensory processing subtypes in autism: Association with adaptive behavior. Journal of Autism and Developmental Disorders, 40, 112–122. 10.1007/s10803-009-0840-2 19644746

[bibr47-13623613211050176] Leekam S. Nieto C. Libby S. J. Wing L. Gould J. (2007). Describing the sensory abnormalities of children and adults with autism. Journal of Autism and Developmental Disorders, 37(5), 894–910. 10.1007/s10803-006-0218-7 17016677

[bibr48-13623613211050176] Leekam S. Prior M. Uljarevic M. (2011). Restricted and repetitive behaviors in autism spectrum disorders: A review of research in the last decade. Psychological Bulletin, 137(4), 562–593. 10.1037/a0023341 21574682

[bibr49-13623613211050176] Leekam S. Tandos J. McConachie H. Meins E. Parkinson K. Wright C. . . . Le Couteur A. (2007). Repetitive behaviours in typically developing 2-year-olds. Journal of Child Psychology and Psychiatry, 48(11), 1131–1138. 10.1111/j.1469-7610.2007.01778.x 17995489

[bibr50-13623613211050176] Lord C. Rutter M. DiLavore P. C. Risi S. Gotham K. Bishop S. (2012). Autism diagnostic observation schedule (2nd ed.). Western Psychological Services.

[bibr51-13623613211050176] Lotan M. Gold C. (2009). Meta-analysis of the effectiveness of individual intervention in the controlled multisensory environment (Snoezelen) for individuals with intellectual disability. Journal of Intellectual and Developmental Disability, 34(3), 207–215. 10.1080/13668250903080106 19681001

[bibr52-13623613211050176] Mey C. Cheng L. Ching L. (2015). The effect of a multisensory program on children with autism. International Journal of Child Development and Mental Health, 3(2), 36–47. https://he01.tci-thaijo.org/index.php/cdmh/article/view/64250

[bibr53-13623613211050176] Mike Ayres Design. (2012). S4.

[bibr54-13623613211050176] Palmer C. Lawson R. Hohwy J. (2017). Bayesian approaches to autism : Towards volatility, action and behaviour. Psychological Bulletin, 143(5), 1–22. 10.1037/bul0000097 28333493

[bibr55-13623613211050176] Pellicano E. Burr D. (2012). When the world becomes ‘too real’: A Bayesian explanation of autistic perception. Trends in Cognitive Sciences, 16(10), 504–510. 10.1016/j.tics.2012.08.009 22959875

[bibr56-13623613211050176] Peterson P. Swing S. Stark K. Waas G. (1984). Students’ cognitions and time on task during mathematics instruction. American Educational Research Association, 21(3), 487–515.

[bibr57-13623613211050176] Pfeiffer B. Coster W. Snethen G. Derstine M. Piller A. Tucker C. (2017). Caregivers’ perspectives on the sensory environment and participation in daily activities of children with autism spectrum disorder. American Journal of Occupational Therapy, 71(4), 1–9. 10.5014/ajot.2017.021360 PMC5490458 28661385

[bibr58-13623613211050176] Pfeiffer B. Erb S. Slugg L. (2019). Impact of noise-attenuating headphones on participation in the home, community, and school for children with autism spectrum disorder. Physical and Occupational Therapy in Pediatrics, 39(1), 60–76. 10.1080/01942638.2018.1496963 30265827

[bibr59-13623613211050176] Powell G. Meredith Z. McMillin R. Freeman T. (2016). Bayesian models of individual differences : Combining autistic traits and sensory thresholds to predict motion perception. Psychological Science, 27(12), 1562–1572. 10.1177/0956797616665351 27770059 PMC5367641

[bibr60-13623613211050176] Reichenbach L. Halperin J. Sharma V. Newcorn J. (1992). Children’s motor activity: Reliability and relationship to attention and behavior. Developmental Neuropsychology, 8(1), 87–97. 10.1080/87565649209540517

[bibr61-13623613211050176] Riddle M. (2002). The Pediatric Anxiety Rating Scale (PARS): Development and psychometric properties. Journal of the American Academy of Child and Adolescent Psychiatry, 41(9), 1061–1069. 10.1097/00004583-200209000-00006 12218427

[bibr62-13623613211050176] Robertson A. Simmons D. (2013). The relationship between sensory sensitivity and autistic traits in the general population. Journal of Autism & Developmental Disorders, 43(3), 775–784. 10.1007/s10803-012-1608-7 22832890

[bibr63-13623613211050176] Robertson A. Simmons D. (2015). The sensory experiences of adults with autism spectrum disorder: A qualitative analysis. Perception, 44(5), 569–586. 10.1068/p7833 26422904

[bibr64-13623613211050176] Rodgers J. Wigham S. McConachie H. Freeston M. Honey E. Parr J. (2016). Development of the anxiety scale for children with autism spectrum disorder (ASC-ASD). Autism Research, 9(11), 1205–1215. 10.1002/aur.1603 26887910

[bibr65-13623613211050176] Rowe C. Candler C. Neville M. (2011). Noise reduction headphones and autism: A single case study. Journal of Occupational Therapy, Schools, and Early Intervention, 4(3–4), 229–235. 10.1080/19411243.2011.629551

[bibr66-13623613211050176] Rutter M. Bailey A. Lord C. (2003). The Social Communication Questionnaire. Western Psychological Services.

[bibr67-13623613211050176] Sayette M. A. Cohn J. F. Wertz J. M. Perrott M. A. Parrott D. J. (2001). A psychometric evaluation of the facial action coding system for assessing spontaneous expression. Journal of Nonverbal Behavior, 25(3), 167–185. 10.1023/A:1010671109788

[bibr68-13623613211050176] Shapiro M. Parush S. Green M. Roth D. (1997). The efficacy of the ‘Snoezelen’ in the management of children with mental retardation who exhibit maladaptive behaviours. British Journal of Developmental Disabilities, 43, 140–155.

[bibr69-13623613211050176] Siper P. M. Kolevzon A. Wang A. T. Buxbaum J. D. Tavassoli T. (2017). A clinician-administered observation and corresponding caregiver interview capturing DSM-5 sensory reactivity symptoms in children with ASD. Autism Research, 10(6), 1133–1140. 10.1002/aur.1750 28296264 PMC8524392

[bibr70-13623613211050176] Spence S. (1999). Spence Children’s Anxiety Scale (parent version). University of Queensland.

[bibr71-13623613211050176] Stephenson J. Carter M. (2011). The use of multisensory environments in schools for students with severe disabilities: Perceptions from teachers. Journal of Developmental and Physical Disabilities, 23(4), 339–357. 10.1007/s10882-011-9232-6

[bibr72-13623613211050176] Unwin K. L. Powell G. Jones C. R. G. (2021). A sequential mixed-methods approach to exploring the experiences of practitioners who have worked in multi-sensory environments with autistic children. Research in Developmental Disabilities, 118, Article 104061. 10.1016/j.ridd.2021.104061 34467871

[bibr73-13623613211050176] Van De Cruys S. Evers K. Van Der Hallen R. Van Eylen L. Boets B. De-Wit L. Wagemans J. (2014). Precise minds in uncertain worlds: Predictive coding in autism. Psychological Review, 121(4), 649–675. https://pdfs.semanticscholar.org/60c6/614872c60b9708488f47521c1c59eda1b9ff.pdf 25347312 10.1037/a0037665

[bibr74-13623613211050176] Wechsler D. (2011). Wechsler Abbreviated Scale of Intelligence, Second edition (WASI-II). NCS Pearson.

[bibr75-13623613211050176] Wechsler D. (2012). Wechsler Preschool and Primary Scale of Intelligence – Forth edition. The Psychological Corp.

[bibr76-13623613211050176] Wigham S. Rodgers J. South M. McConachie H. Freeston M. (2015). The interplay between sensory processing abnormalities, intolerance of uncertainty, anxiety and restricted and repetitive behaviours in autism spectrum disorder. Journal of Autism and Developmental Disorders, 45, 943–952. 25261248 10.1007/s10803-014-2248-x

